# Secondary surgery and mortality following primary treatment for acetabular fractures – an observational study from Swedish national quality registers

**DOI:** 10.1186/s13018-025-05796-y

**Published:** 2025-05-16

**Authors:** Madelene Albrektsson, Michael Möller, Mikael Sundfeldt, David Wennergren, Olof Wolf

**Affiliations:** 1 Department of Orthopaedics, Institute of Clinical Sciences, Sahlgrenska Academy, University of Gothenburg, Gothenburg, Sweden; 2 Department of Orthopaedics, Sahlgrenska University Hospital, Gothenburg, Mölndal Sweden; 3 Department of Surgical Sciences, Uppsala University, Uppsala, Sweden; 4 Department of Orthopaedics and Hand Surgery, Uppsala University Hospital, Uppsala, Sweden

**Keywords:** Acetabular fractures, Secondary treatment, Reoperations, Revision surgery, Total hip arthroplasty, Mortality, Swedish fracture register

## Abstract

**Background:**

The treatment of acetabular fractures ranges from non-operative with no restrictions in mobilisation to some of the more complex operative treatments in orthopaedics. Treatment strategies are developing, and outcomes need to be studied continuously. The study’s primary aim was to assess the rate of secondary treatment in patients with acetabular fractures treated non-operatively or operatively. A secondary aim was to study mortality.

**Methods:**

Data were retrieved from the Swedish Fracture Register and cross-referenced with the Swedish Arthroplasty Register for all patients aged ≥ 18 years with an acetabular fracture between 2014 and 2023. Patients were divided into three primary treatment groups: non-operative treatment, open reduction and internal fixation (ORIF), and total hip arthroplasty (THA) with/without combined ORIF (THA/combined hip procedure, CHP). The study examined mortality rates within each treatment group.

**Results:**

Of the 3318 patients included in the study, 74% were treated non-operatively, 18% with ORIF, and 8% with THA/CHP. 4% of non-operatively treated patients and 17% of patients treated with ORIF had been converted to THA at 5 years, 12% of patients with THA as primary treatment had been revised. Patients who underwent THA as their initial treatment were more likely to undergo secondary treatment early. However, in those initially treated with ORIF the prevalence of secondary treatment increased after the first year. The non-operatively treated group had the highest mortality rate (19% at 1 year), followed by the THA group (14% at 1 year).

**Conclusions:**

This observational nationwide register study on acetabular fractures shows that surgically treated patients have a relatively high reoperation rate. Younger patients are predominately treated with ORIF and display low mortality rates. Older patients with complex fracture patterns may benefit from primary treatment with THA/CHP being more frequently performed compared to prevailing practice.

## Background

Acetabular fractures have a profound impact on individuals of all age groups, leading to life-altering consequences and persisting impairments in both function and mobility. Patients sustaining acetabular fractures are often divided into two main groups: The younger population who suffers high-energy trauma and the older population who sustains an acetabular fracture resulting from a simple fall [1]. Treatment options differ between these patient groups. Younger patients with displaced fractures are more often treated with open reduction and internal fixation (ORIF). Older and frailer patients may encounter technical difficulties with ORIF due to comminution and poor bone quality, resulting in discouraging outcomes for this specific patient demographic [2–5]. For many older patients with acetabular fractures, non-operative treatment is a viable and commonly pursued option. Still, an active patient’s more complex and displaced fractures also need stabilisation at a higher age [6]. Primary total hip arthroplasty (THA) alone or in combination with ORIF (known as combined hip procedure, CHP) has become a more frequent treatment option for this patient group [7–9].

This observational register study used Swedish national quality registers to assess the rate of secondary treatment after either non-operative primary treatment or operative primary treatment with ORIF or THA/CHP for acetabular fracture patients. Due to the rising incidence of acetabular fractures in the elderly with a population that is becoming increasingly similar to hip fracture patients with a well-known increased risk of death, mortality within each treatment group was investigated as a secondary aim [1, 10].

## Methods

### Selection criteria

**Fig. 1:**
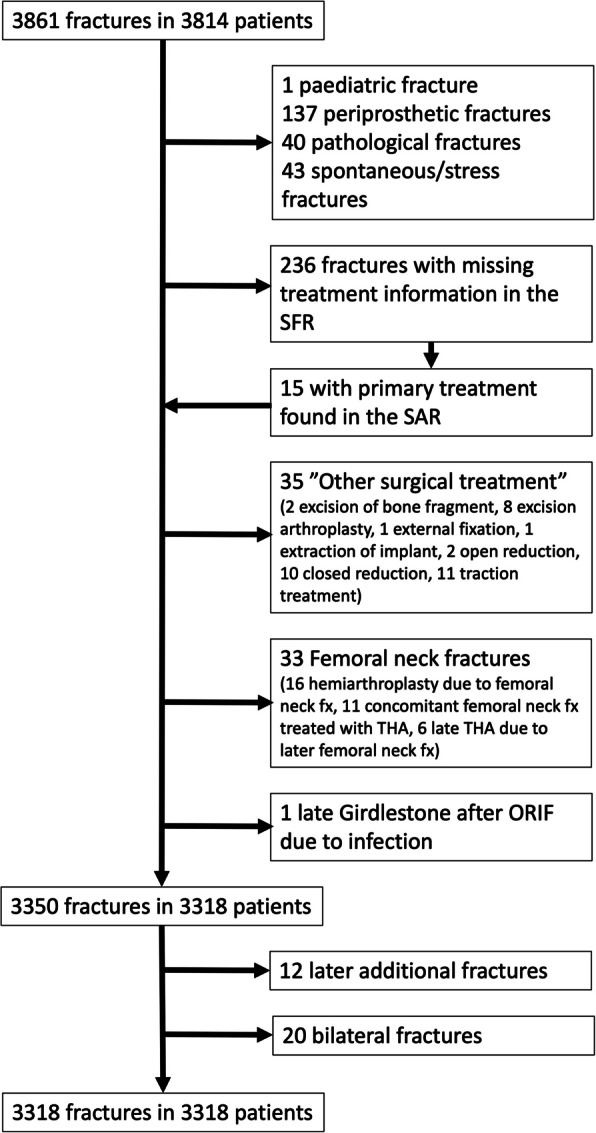
Flowchart of patients included in the study. SFR – Swedish Fracture Register, SAR – Swedish Arthroplasty Register, ORIF – Open Reduction Internal Fixation, fx – fracture

Data on all patients ≥ 18 years old at the time of injury with an acetabular fracture registered from January 1, 2014 to October 18, 2023 were retrieved from the Swedish Fracture Register (SFR). This cohort was cross-referenced with the Swedish Arthroplasty Register (SAR), and information on both primary and secondary treatment was retrieved to add information to the SFR data. Patients were divided into three primary treatment groups: non-operative, ORIF and primary THA/CHP. The primary data set from the SFR contained 3861 fractures in 3814 patients. Patients with other primary treatment methods or missing treatment information were excluded (Fig. 1). Moreover, individuals who had periprosthetic, pathological, stress, or paediatric fractures, as well as those who received arthroplasty for a femoral neck fracture, were excluded from the study. After these exclusions, 3350 fractures in 3318 patients were eligible for the study. To avoid dependency issues for Kaplan Meier and mortality analysis, only one fracture per patient was included in the final study cohort [11]. Only one fracture was included in the analysis among the 20 patients with simultaneous bilateral fractures. Only the first fracture was included for 12 patients with a subsequent acetabular fracture (nine contralateral, four ipsilateral) during the inclusion period. The final study cohort was 3318 fractures in 3318 patients.

When multiple early treatment registrations were found in the registers, a primary THA within 6 weeks of the injury date was considered primary treatment, which allowed a staged CHP to be included in case of for example perioperative bleeding during ORIF, or patient to be resuscitated before THA after ORIF. In the absence of a primary THA registration in the same period an ORIF registration within 6 weeks of injury was determined as the primary treatment, and later THA defined as a reoperation due to symptomatic complaints.

### Study variables

Variables extracted from the SFR encompassed age, sex, injury mechanism and date, fracture type, primary treatment, and date of death. Each individual was cross-reference with the SAR using their unique personal identification number (PIN) to identify secondary treatment, i.e. conversion to THA after primary ORIF or non-operative treatment, or revision surgery after primary THA treatment.

### Study outcomes

Secondary treatment, as defined above, was the primary outcome. A secondary outcome was mortality, with comparison between treatment groups. The end of follow-up was set at the time of data extraction from the register (November 27, 2023).

### Statistical methods

Descriptive statistics were used for baseline variables and presented as medians (range) and proportions. Survival analyses depicting 1) secondary treatment, and 2) mortality were performed using Kaplan–Meier estimates with 95% confidence intervals (CIs). Censoring was the end of follow-up or death, whichever occurred first. Unadjusted cumulative 1) secondary treatment, and 2) mortality rates with 95% CIs were estimated using the Kaplan–Meier method. The association between 1) secondary treatment and 2) mortality and primary treatment (non-operative, ORIF, or THA/CHP), adjusted for age and sex, were examined using a Cox regression model. The calculation and plotting of Schönfeld residuals were conducted to verify the underlying assumptions of the Cox regression model.

SPSS Statistics (version 29, IBM Corporation, USA) and R v4.3.1 (R Foundation for Statistical Computing, Vienna, Austria) were used for statistical analyses.

### Ethical approval

The Swedish Ethical Review Authority (registration numbers 2020–03775 and 2023–01499-02) granted ethical approval.

## Results

Of the 3318 fractures in 3318 patients included in the study, 2468 (74%) were treated non-surgically, 585 (18%) with ORIF and 265 (8%) received primary THA/CHP. Sex, age, and fracture classifications for the three primary treatment groups are shown in Table 1. Men accounted for 64% of the total fractures. The ORIF group was predominantly male, with men accounting for 79% of the patients. The median age was 59 years. The THA/CHP group had a median age of 78 years, and the non-operatively treated group had a median age of 79 years.

**Table 1:**
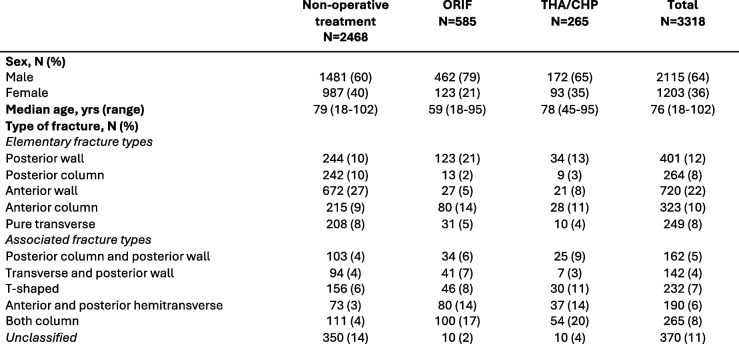
Demographics of 3318 patients with acetabular fractures (sex, age, and fracture classification) by primary treatment

*ORIF *Open reduction internal fixation, *THA *Total hip arthroplasty, *CHP *Combined hip procedure

### Secondary treatment

**Fig. 2:**
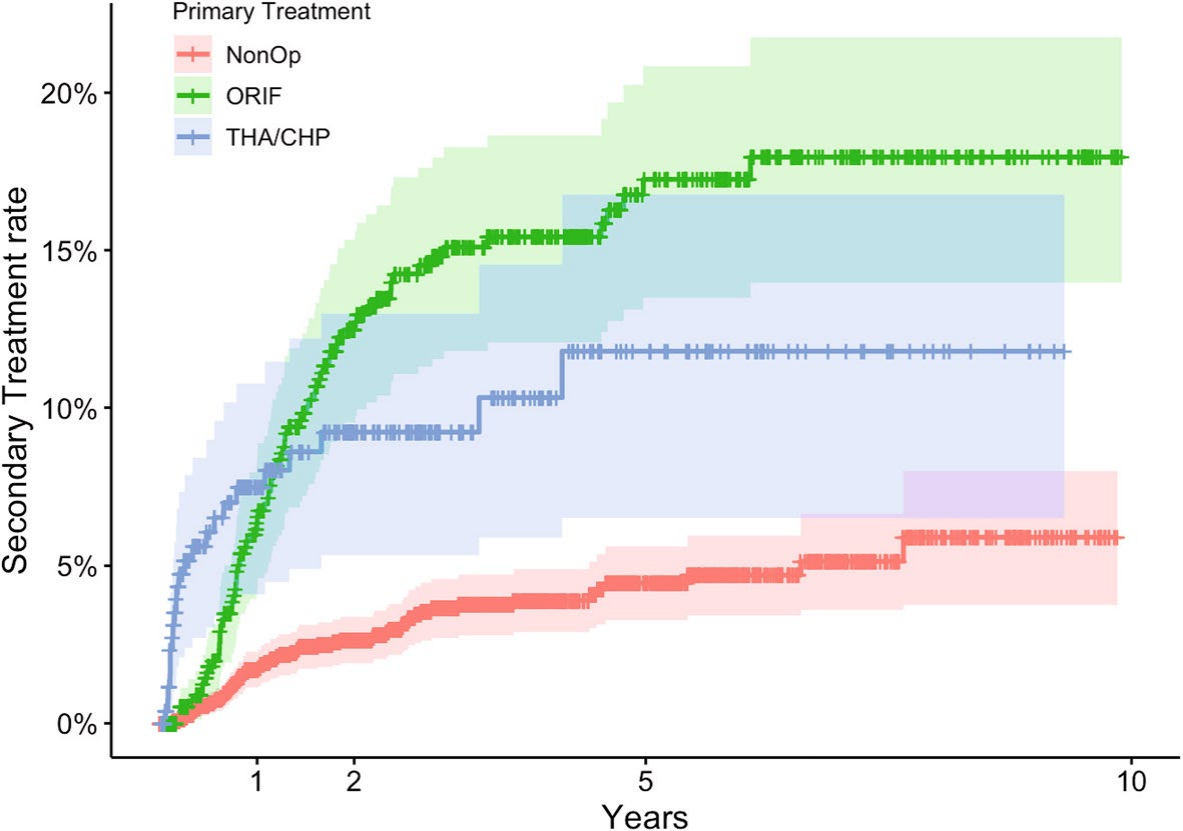
Secondary treatment rates up to 10 years after fracture for patients with acetabular fracture treated non-operatively, with ORIF, or with THA/CHP. Kaplan–Meier survival curve with 95% confidence intervals. NonOp – Non-operative treatment. ORIF – Open reduction and internal fixation. THA/CHP – Total hip arthroplasty/Combined hip procedure

At 1 year, the cumulative secondary treatment rate was 1.7% (CI 1.1–2.3) for the non-operative group, 6.2% (CI 4.1–8.2) for ORIF, and 7.5% (CI 4.1–10.8) for THA/CHP (Fig. 2 and Table 2). At 5 years, secondary treatment rates were 4.4% (CI 3.3–5.6), 17.3% (CI 13.5–20.8), and 11.8% (CI 6.5–16.8) for the respective groups. The Kaplan–Meier survival curve shows that most secondary procedures are performed within the first year for the THA/CHP group and within the first 2 years for the ORIF group. In the non-operatively treated group, the occurrence of joint failure leading to late THA develops gradually over the 10-year follow-up period with approximately 5% requiring secondary intervention. However, no statistically significant long-term differences were observed between the ORIF and THA/CHP groups.

**Table 2:** Unadjusted secondary treatment rates (95% CI) at 1, 2, and 5 years for patients with acetabular fractures primarily treated non-operatively, with ORIF, or with THA/CHP

	**Non-operative treatment **% (95%CI)	**ORIF **% (95%CI)	**THA/CHP **% (95%CI)
1 year	1.7 (1.1–2.3)	6.2 (4.1–8.2)	7.5 (4.1–10.8)
2 years	2.6 (1.9–3.4)	12.5 (9.5–15.3)	9.2 (5.3–13.0)
5 years	4.4 (3.3–5.6)	17.3 (13.5–20.8)	11.8 (6.5–16.8)

*ORIF* Open reduction internal fixation, *THA *Total hip arthroplasty, *CHP *Combined hip procedure

Cox regression analysis revealed a higher hazard ratio (HR) for secondary treatment in both the ORIF (HR 4.4, CI 3.1–6.3), and THA/CHP groups (HR 3.3, CI 2.0–5.3) compared to the non-operative group, adjusted for age and sex.

The secondary treatment rate among patients primarily treated non-operatively was highest for associated fracture types and for posterior wall fractures (Table 3). When primarily treated with ORIF, the associated posterior column and posterior wall fractures had the highest secondary treatment rates, followed by T-shaped and elementary posterior wall fractures. Secondary treatment rates after THA/CHP were highest for the pure transverse and the transverse and posterior wall fractures.

**Table 3:** Secondary treatment rates, expressed in percentage (%), per fracture type for patients with acetabular fractures primarily treated non-operatively, with ORIF, or with THA/CHP

	**Non-operative treatment %**	**ORIF %**	**THA/CHP %**
*Elementary fracture types*			
Posterior wall	4.9	17.1	8.8
Posterior column	1.2	15.4	11.1
Anterior wall	1.6	11.1	0.0
Anterior column	1.9	12.5	10.7
Pure transverse	3.8	9.7	30.0
*Associated fracture types*			
Posterior column and posterior wall	6.8	17.6	8.0
Transverse and posterior wall	3.2	14.6	28.6
T-shaped	5.1	17.4	10.0
Anterior and posterior hemitransverse	5.5	12.5	8.1
Both column	4.5	10.0	1.9
* Unclassified*	0.9	0.0	20.0

*ORIF* Open reduction internal fixation, *THA* Total hip arthroplasty, *CHP* Combined hip procedure

### Mortality

**Fig. 3:**
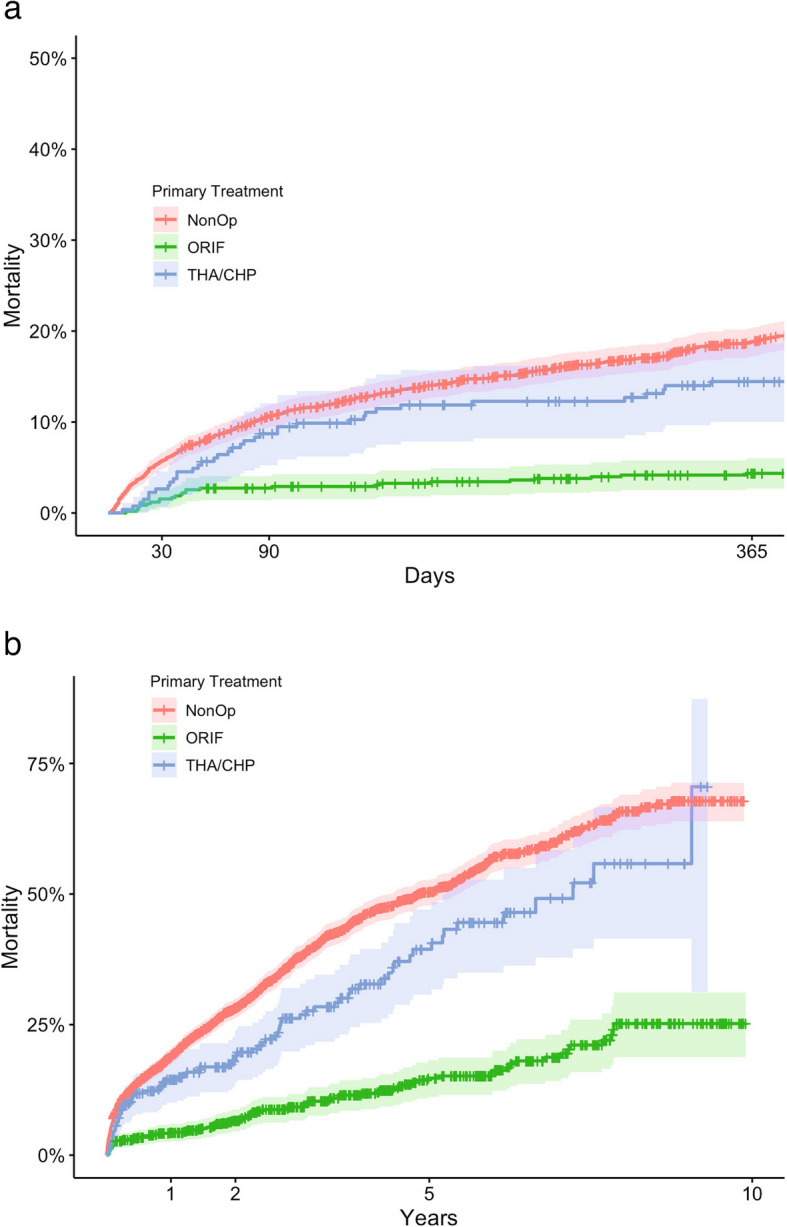
**a** Unadjusted cumulative mortality up to 1 year for patients with an acetabular fractures depending on primary treatment (non-operative, ORIF, or THA/CHP). Kaplan–Meier survival curves with 95% confidence intervals. **b** Unadjusted cumulative mortality up to 10 years for patients with an acetabular fracture depending on primary treatment (non-operative, ORIF, or THA/CHP). Kaplan–Meier survival curves with 95% confidence intervals. NonOp – Non-operative treatment. ORIF – Open reduction and internal fixation. THA/CHP – Total hip arthroplasty/Combined hip procedure

The unadjusted crude 30-day mortality was 5.7% (CI 4.8–6.6) in patients treated non-operatively, 1.5% (CI 0.2–2.5) in ORIF-treated patients and 2.6% (CI 0.7–4.6) in patients primarily treated with THA/CHP (Fig. 3 and Table 4). After 1 year, mortality rates were 18.8% (CI 17.2–20.4), 4.4% (CI 2.7–6.0), and 14.4% (CI 10.0–18.7) for the respective treatment groups.

**Table 4:** Unadjusted mortality rates (95% CI) at 30 days, 3 months, and 1 year for patients with acetabular fractures primarily treated non-operatively, with ORIF, or with THA/CHP

	**Non-operative treatment **% (95%CI)	**ORIF **% (95%CI)	**THA/CHP **% (95%CI)
30 days	5.7 (4.8–6.6)	1.5 (0.5–2.5)	2.6 (0.7–4.6)
3 months	10.7 (9.5–11.9)	2.7 (1.4–4.0)	8.7 (5.2–12.0)
1 year	18.8 (17.2–20.4)	4.4 (2.7–6.0)	14.4 (10.0–18.7)

*ORIF* Open reduction internal fixation, *THA* Total hip arthroplasty, *CHP* Combined hip procedure

Cox regression analysis showed a lower HR for death for both the ORIF (HR 0.6, CI 0.5–0.8), and the THA/CHP (HR 0.7, CI 0.6–0.9) groups when compared to the non-operative group, adjusted for age and sex.

Mortality in the ORIF group was significantly lower at all time points, except compared to THA/CHP at 30 days.

## Discussion

The majority of patients with acetabular fractures in this national observational register study were treated non-operatively. The comparison included three main treatment methods. The ORIF group is younger, and nearly four out of five patients are men.

### Secondary treatment

Secondary treatment was the least common for patients with initial non-operative treatment. This finding can have several explanations but suggests that the indications for non-operative treatment are reasonable in this population. The treatment method is based on multiple factors including fracture type and displacement, the patient’s functional demand, and comorbidities. The small number of non-operatively treated patients who underwent secondary treatment with later THA can be attributed to either a simple, undisplaced fracture that heals without complications, or a frail patient unsuitable for any surgery either early or at a later stage. In a review by Cacciola et al. the overall conversion rate to THA after non-operative treatment of acetabular fractures in patients ≥ 60 years was 8.3%, slightly higher than the rate observed in the current study [6].

Patients who underwent ORIF as their initial treatment had the highest rate of secondary treatment with conversion to arthroplasty in long-term follow up (12.5% at 2 years and 17.3% at 5 years). These patients have undergone an intervention to preserve the integrity of the native hip. Hence, they are at risk of developing post-traumatic arthritis or necrosis of the femoral head, resulting in the need for late THA surgery. Previous studies have reported the rate of late THA after primary ORIF to be 13–21% [12–16]. Other complications such as heterotopic ossification and postoperative infections add to patients’ residual dysfunction and prolonged hospital stay. Although follow-up times differ between studies, the current national observational study suggests comparable or slightly lower rates of late THA in the ORIF group. The reason for a lower national rate could be attributed to the centralisation of most ORIF cases in Sweden to university hospitals.

Given that the selection of ORIF-treated patients is based on surgical indications, it is crucial to determine which patients are at the highest likelihood of requiring a THA within the initial years following surgery. Tannast et al. identified patient age over 40 years, marginal impaction, posterior wall involvement, femoral head lesions, and an initial articular displacement of 20 mm or more to have a negative impact of the survival of the native hip [17]. Prior to surgery, it is essential to provide comprehensive preoperative counselling to patients with fracture configurations that pose a high risk of early conversion. This counselling should address the potential risks and benefits associated with various treatment options, taking into account the patient’s age. Unless the patient is quite young, it may be more advisable to prioritise treatment with a THA/CHP to minimise unnecessary surgeries and patient distress. Consistent with previous studies, complex fracture patterns and fractures involving the posterior wall have been associated with a poor prognosis, leading to a higher rate of late THA in both non-operatively and ORIF-treated patients [14, 18, 19]. Extra care should be taken when assessing and determining treatment strategies for these patients.

The study revealed that when THA/CHP was the primary treatment, 7.5% of patients with acetabular fractures necessitated secondary treatment within the first year after the injury. This percentage is similar or lower compared to other studies on acute THA but higher than elective THA due to coxarthrosis, where the revision rate within 2 years is approximately 2% in Sweden [12, 13, 20]. Previous studies have shown that primary THA/CHP has a higher risk of hip dislocation and postoperative infection compared to elective THA due to suboptimal circumstances in the acute setting, such as preoperative preparations and soft tissue injuries [21]. Nevertheless, the long-term findings of the present study indicate a tendency towards reduced rates of secondary treatment for THA/CHP compared to the ORIF treatment group. Secondary treatment with arthroplasty procedures comes earlier in the THA/CHP group, probably because infection and dislocation are indications compared to the ORIF group, where indications are postoperative joint failure. Acute THA in acetabular fracture patients has also been shown to have lower revision rates compared to late THA following failed ORIF [22]. THA/CHP as primary treatment for acetabular fracture patients has increased in popularity due to encouraging results, especially for the osteoporotic patients with certain fracture patterns such as impaction of the joint surface, comminution of the posterior wall and preexisting hip osteoarthritis [23–25]. Our study further supports this concept, suggesting that through careful selection, improved surgical techniques, and expertise, patients with acetabular fractures who undergo acute THA/CHP may be able to avoid subsequent surgeries.

### Mortality

Mortality in the three treatment groups differed significantly. This mortality difference is likely attributable to selection bias rather than the treatment itself. The non-operatively treated group had the highest mortality rate, comparable to hip fracture patients. However, this group had the highest median age and can be assumed to have more comorbidities [10]. The THA/CHP group had a marginally lower mortality rate despite comparable age and sex distributions to the non-operative group. They were, however, selected to undergo major surgery and were likely less comorbid and much healthier. The ORIF group, which comprised the youngest and presumably the healthiest individuals, had the lowest mortality rate. The operative groups had a lower HR than the non-operative group even after adjusting for age and sex, suggesting that other factors might contribute, i.e. residual confounding. The healthier patients were more prone to receive operative treatment, and we could not adjust for comorbidities in this data set.

### Strengths and limitations

This study has certain limitations. The coverage and completeness have increased since the SFR’s introduction in 2011 because of Sweden’s stepwise affiliation of orthopaedic departments. In 2020 SFR reached 100% coverage of orthopaedic departments [26]. Completeness was 42% for pelvic and acetabular fractures in 2022 compared to data from the National Patient Register, which is recognised for its inflated rates [27]. This low completeness is probably mostly due to non-operatively treated rami fractures not being registered. SFR data on secondary treatment are known to have limited completeness [28, 29]. To overcome this all patients were cross-referenced with the SAR, which have completeness rates for primary THA of 98% in 2021 and 94% for hip revisions [30]. Outcomes were defined as secondary treatment with arthroplasty procedures. Although other secondary operative therapies could have been performed in a small group of patients, we feel confident that our outcomes best capture joint failure or failure of primary THA.

Despite missing patients in the registers, our cohort is comparatively large, with excellent control of the primary outcome, allowing relevant analysis and conclusions to be drawn. Additionally, although the fracture classifications in SFR have been validated, the correctness differs between fracture types, which needs to be considered when interpreting results that entail comparing fracture types [31].

In conclusion, one in five patients treated with ORIF for acetabular fracture have undergone an arthroplasty procedure at 5 years compared to only 4% having been converted after non-operative primary treatment. Current patient selection for treatment of acetabular fractures in Sweden is reasonable, wherein younger patients undergo ORIF, while older adults are either managed non-operatively or receive THA/CHP treatment. Nonetheless, there is potential for refining patient selection in the subset currently undergoing ORIF treatment. To minimise the need for additional surgeries, primary THA/CHP is recommended as a more extensive treatment option for older healthy adults with complex acetabular fractures assessed to need operative treatment. This suggestion is supported by the fact that the larger primary procedure does not result in increased adjusted HRs compared to ORIF.

## Data Availability

The data analysed in this study is not publicly available due to Swedish legislation on public access and secrecy. The study was approved on the grounds of ensuring the confidentiality of patient-identifiable information. After ethical approval from the Swedish Ethical Review Authority, individuals interested in this dataset can apply to retrieve data from the Center of Registers, Västra Götaland, Sweden (URL: http://registercentrum.se/).
